# Impact of β-Amyloids Induced Disruption of Ca^2+^ Homeostasis in a Simple Model of Neuronal Activity

**DOI:** 10.3390/cells11040615

**Published:** 2022-02-10

**Authors:** Francisco Prista von Bonhorst, David Gall, Geneviève Dupont

**Affiliations:** 1 Unit of Theoretical Chronobiology, Faculté des Sciences (CP231), Université Libre de Bruxelles (ULB), 1050 Brussels, Belgium; francisco.prista.santos.von.bonhorst.silva@ulb.be; 2 Research Laboratory in Human Reproduction, Faculté de Médecine (CP636), Université Libre de Bruxelles (ULB), 1070 Brussels, Belgium; david.gall@ulb.be

**Keywords:** Ca^2+^, dynamics, Alzheimer’s disease, computational model, bifurcation analysis

## Abstract

Alzheimer’s disease is characterized by a marked dysregulation of intracellular Ca^2+^ homeostasis. In particular, toxic β-amyloids (Aβ) perturb the activities of numerous Ca^2+^ transporters or channels. Because of the tight coupling between Ca^2+^ dynamics and the membrane electrical activity, such perturbations are also expected to affect neuronal excitability. We used mathematical modeling to systematically investigate the effects of changing the activities of the various targets of Aβ peptides reported in the literature on calcium dynamics and neuronal excitability. We found that the evolution of Ca^2+^ concentration just below the plasma membrane is regulated by the exchanges with the extracellular medium, and is practically independent from the Ca^2+^ exchanges with the endoplasmic reticulum. Thus, disruptions of Ca^2+^ homeostasis interfering with signaling do not affect the electrical properties of the neurons at the single cell level. In contrast, the model predicts that by affecting the activities of L-type Ca^2+^ channels or Ca^2+^-activated K^+^ channels, Aβ peptides promote neuronal hyperexcitability. On the contrary, they induce hypo-excitability when acting on the plasma membrane Ca^2+^ ATPases. Finally, the presence of pores of amyloids in the plasma membrane can induce hypo- or hyperexcitability, depending on the conditions. These modeling conclusions should help with analyzing experimental observations in which Aβ peptides interfere at several levels with Ca^2+^ signaling and neuronal activity.

## 1. Introduction

Ca^2+^ ions play a key role in intracellular signaling by controlling vital physiological processes such as secretion, gene expression, and apoptosis [1]. In neurons, they also act as a charge carrier. Thus, during neuronal activity, Ca^2+^ concentration and plasma membrane (PM) voltage changes are intimately related: Ca^2+^ entry through voltage gated Ca^2+^ channels can affect Ca^2+^ signaling, while voltage independent Ca^2+^ changes are able to alter PM excitability [2].

This dual role of Ca^2+^ as an ion involved in signaling and in neuronal activity is highly relevant for the understanding of the development of neurodegenerative diseases and, in particular, Alzheimer’s disease (AD). Together with the hyperphosphorylation of τ proteins and the accumulation of amyloid-β peptides (Aβ) inside neurons, the progression of AD is indeed characterized by a perturbation of intracellular Ca^2+^ homeostasis [3]. Ca^2+^ dysregulation associated with neurodegenerative diseases is multifactorial. Aβ can indeed affect Ca^2+^ exchanges with the extracellular medium and with internal Ca^2+^ stores [4]. These modifications result in an increase in cytosolic Ca^2+^. It is now widely accepted that disruption of ER calcium mediates the most significant signaling pathways that are associated with Alzheimer’s disease, and is a main driving force in the development of the disease [5,6,7]. Among many consequences, excessive Ca^2+^ increase can lead to apoptotic neuronal death. Ca^2+^ also stimulates the activity of γ-secretase, a key enzyme involved in the production of toxic Aβ peptides. Such a vicious circle between amyloids and Ca^2+^ has been proposed to play a key role in the progressive and irreversible character of the disease [4,8].

At the level of neuronal activity, extracellular plaques of Aβ peptides are reported to induce electrical hyperexcitability [9]. Such an excitatory effect relies on a network effect associated with the disruption of synaptic function [6], but similar observations have been found on cultured neurons [10] or in brain slices [11,12]. How Aβ peptides, Ca^2+^, and neuronal excitability are related to each other at the single cell level is not well understood and is difficult to assess experimentally. Intuitively, increases in Ca^2+^ are expected to be associated with a decrease in neuronal activity via the activation by Ca^2+^ of a hyperpolarizing K^+^ current mediated by SK or BK channels, which represent the major link between Ca^2+^ concentration and the electrical activity of the plasma membrane. The question thus arises as to how AD’s progression can be associated at the same time with the increase in intracellular Ca^2+^ that follows the calcium hypothesis of AD, and with an increase in neuronal electrical activity. Computational modeling is well suited to gain insight into this paradox, because it allows for individually assessing the consequence of Aβ peptides on their various targets. It can also predict their relative importance.

From a mathematical point of view, interactions between membrane electrical activity and Ca^2+^ signaling have been described in specific cell types, such as astrocytes [13,14], pancreatic cells [15,16], GnRH neurons [17] motoneurons [18], and cardiac cells [19,20]. To the best of our knowledge, there are, however, very few general computational models describing electrical activity and intracellular Ca^2+^ dynamics in a prototypical neuronal cell. An early generic model of a neuron shows that less current is necessary to generate an AP when Aβ peptides are considered to block the fast-activating potassium current [21]. In a previous study, we used a simple model of the electrical activity of granule cells, considering its interplay with membrane Ca^2+^ dynamics, to study the impact of tonic NMDA receptor activity on neuronal excitability [22]. Modeling could show that the constant Ca^2+^ influx induced by this tonic activity can lock previously active neurons in a hyperactive state. This result agrees with the observed hyperactivation of neurons in presence of Aβ-induced glutamate spill-over [23,24].

In the present study, we extend the same minimal model of granule cell electrical activity and membrane Ca^2+^ dynamics to consider a more accurate description of the evolution of intracellular Ca^2+^, including exchanges with the ER. The model involves three compartments: a fictious, sub-plasmalemmal shell that controls PM electrical activity, the cytosol, and the ER. It considers Ca^2+^ fluxes through the plasma membrane Ca^2+^ ATPase (PMCA), the sodium calcium exchanger (NCX), the sarco- or endoplasmic reticulum Ca^2+^ ATPase (SERCA) pumps, and the inositol 1,4,5-trisphosphate receptor (IP_3_) and ryanodine receptors (IP_3_R and RYR), grouped together in a single phenomenological flux. In this first approach, we indeed do not incorporate Ca^2+^ fluxes resulting from stimulation by IP_3_-inducing agonists. Our aim is to investigate how long-term alterations in Ca^2+^ fluxes modify the resting concentrations of Ca^2+^ in the different compartments and neuronal excitability. The model is then used to systematically investigate the various effects of Aβ peptides that have been reported in the literature. We found a strong independence between changes in cytosolic and subplasmalemmal Ca^2+^ concentrations, allowing cytosolic Ca^2+^ to vary without affecting neuronal excitability. In contrast, the latter is much affected by changes in subplasmalemmal Ca^2+^, with decreases in PMCA activity provoking neuronal hypo-excitability. Hyperexcitability is however predicted by the model when simulating the Aβ peptides reported changes in the L-type Ca^2+^ current [25] and the experimentally reported effect of Aβ peptides on Ca^2+^-sensitive K^+^ channels [26]. Lastly, an influx of Ca^2+^ flux through the pores of Aβ oligomers [27,28] can also increase the frequency of the simulated spikes. Altogether, the model helps understanding the apparent paradoxical dual role of Aβ peptides in increasing intracellular Ca^2+^ and provoking neuronal hyperexcitability. It also provides a simple description of intracellular Ca^2+^ dynamics and membrane excitability that can be further adapted to describe Ca^2+^ dysregulation in neurodegenerative diseases in a finely tuned manner.

## 2. Description of the Model

The core model used in this study is a previously proposed simple mathematical description of the electrical activity of cerebellar granule cells [2,29]. This model has been experimentally validated and can be seen as a good description of the electrical activity of a prototypical neuron. In the context of Alzheimer’s disease, we extended the model to consider tonic activity of NMDA receptors [22], which results from the Aβ-induced spill-over of glutamate [30].

**Figure 1:**
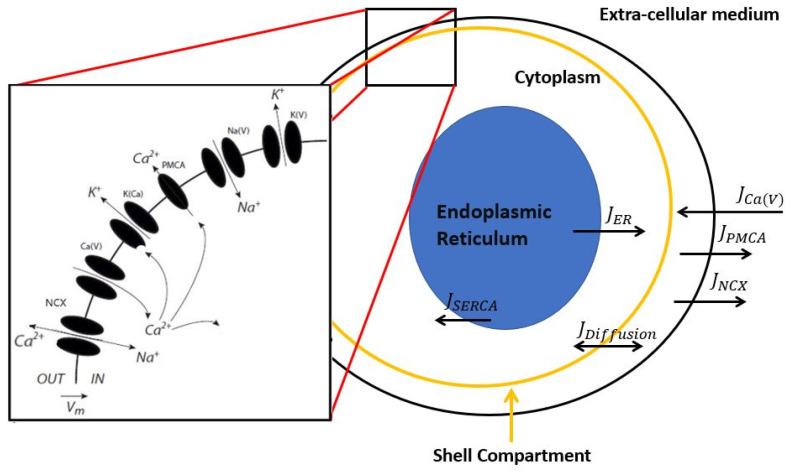
Schematic representation of the simple, general model of neuronal activity and Ca^2+^ dynamics in the absence of IP_3_-generating agonist. The currents that directly govern membrane electrical activity are represented in the black square and correspond to high-voltage gated calcium channels (Ca(*V*)), calcium-activated potassium channels (K(Ca)), voltage-dependent sodium channels (Na(*V*)), delayed rectifier potassium channels (K(*V*)), and the sodium–calcium exchanger (NCX). Except for the latter, the formulation of all these currents is taken directly from Gall et al., 2003. In the present study, we consider three Ca^2+^ compartments, corresponding to the sub-plasmalemmal space, the bulk of the cytosol, and the ER. When active, the voltage-gated calcium channels transport Ca^2+^ in the sub-plasmalemmal space. From this compartment, Ca^2+^ is transported outside the cell via PMCA and NCX. Exchanges between the cytosol and ER are mediated by the SERCA and the IP_3_R and RyR that are gathered in a *J_ER_* flux because we do not consider the situation in which the cell would be stimulated by an IP_3_- or NAADP-generating agonist.

The initial core model describes the evolution of the membrane voltage and Ca^2+^ concentration in a sub-plasmalemmal compartment that can be seen as a thin shell just below the plasma membrane (*Ca*). The currents included in the model, schematized in black in Figure 1, are a voltage-dependent Na^+^ current (*I_Na_*_(*V*)_), a delayed rectifier K^+^ current (*I_K_*_(*V*)_), a high-threshold voltage dependent Ca^2+^ current (*I_Ca_*_(*V*)_), a Ca^2+^-activated K^+^ current (*I_K_*_(*Ca*)_), and *I_inj_* is the current injected into the cell that allows for studying its excitability properties. The mathematical expressions used for the currents, together with the evolution equations for the gating variables, are given in the Supplementary Material. Only one type of *I_K(Ca)_* current is considered explicitly. In reality, most excitable cells express both large-conductance (BK) and small-conductance (SK) calcium-activated potassium channels. The former is sensitive to Ca^2+^ only, while the latter is sensitive to both voltage and calcium. For simplicity, Equations (S7), (S18) and (S19) empirically describe the two types of channels. The equations and parameter values that govern the dynamics of opening/closing of the ion channels are the same as in the previous studies [2], and are given in Supplementary Material and Table 1. Temporal evolution of the membrane voltage is as follows:

(1) $$C_{m}\frac{dV}{dt}=-I_{Na\left(V\right)}-I_{K\left(V\right)}-I_{Ca\left(V\right)}-I_{K\left(Ca\right)}+I_{Inj}$$

where *C_m_* is the cell capacitance. When active, the *I_Ca_*_(*V*)_ current mediates the entry of Ca^2+^ into the cell and hence leads to an increase in Ca^2+^ concentration in the sub-plasmalemmal compartment. Thus,

(2) $$\frac{dCa}{dt}=f\left[\frac{-I_{Ca\left(V\right)}}{2FV_{shell}}-\beta \;Ca\right]$$

where *f* is the buffering capacity of the cytoplasm, *F* the Faraday constant, *V_shell_* the volume of the sub-plasmalemmal compartment, and *β* is a first order rate constant that empirically gathers all the processes that remove Ca^2+^ from the shell.

Here, we extend the model to consider a cytosolic and an endoplasmic reticulum (ER) Ca^2+^ compartment, and to provide a more accurate description of the processes that remove Ca^2+^ out of the shell. Note that while the subplasmalemmal space represents a fictious compartment, the cytosol and ER correspond to real cellular spaces delimited by membranes. The model is schematized in Figure 1. From the sub-plasmalemmal compartment, Ca^2+^ is extruded towards the extracellular medium via an ATPase and via the Na^+^/Ca^2+^ exchanger (NCX). The rate of Ca^2+^ transport through the ATP-consuming plasma membrane Ca^2+^ ATPase (PMCA) is given by:

(3) $$J_{PMCA}=V_{PMCA}\frac{Ca^{2}}{K_{PMCA}^{2}+Ca^{2}}$$

in which *V_PMCA_* and *K_PMCA_* stand for the maximal velocity and the half saturation constant of the PMCA, respectively. Note that export of Ca^2+^ by the PMCA is accompanied by a concomitant influx of H^+^ [31], in such a way that the activity of this pump does not influence the membrane voltage. The Na^+^/Ca^2+^ exchanger removes Ca^2+^ from the cytoplasm at the expense of Na^+^ entry, with a 1:3 stoichiometry. It is thus electrogenic. Following Gall et al. (1999) [16], we describe this current by

(4) $$I_{NCX}=g_{NCX}\frac{Ca^{2}}{K_{NCX}^{2}+Ca^{2}}\left(V-V_{NCX}\right)$$

where *g_NCX_* is the NCX conductance, *K_NCX_* the half saturation constant for NCX activation by Ca^2+^, and *V_NCX_* the resting potential for this channel calculated using resting Na^+^ and Ca^2+^ concentrations. This current is added (with a negative sign) to the right-hand side of Equation (1) and with the appropriate factors to the evolution equation for *Ca* (see below).

To introduce Ca^2+^ diffusion from the sub-membrane region to the cytosol, we constructed a compartmental model wherein the cytoplasm and the shell are assumed to be homogeneous compartments. In such an approach, the corresponding efflux of Ca^2+^ from the cytosol considers the Ca^2+^ diffusion coefficient and the respective volumes of the compartments. It can be written as:

(5) $$J_{diff}=-\gamma \left(Ca-Ca_{cyt}\right),\;\mathrm{with}\;\gamma =\frac{D}{h^{2}}$$

where *D* stands for the effective Ca^2+^ diffusion in the cytosol and *h* for the distance between the shell and the cytoplasmic compartment. Thus, for a typical granule cell with a radius of ~4 μm, *γ* is around 0.001 ms^−1^ considering that *D* = 15 μm^2^s^−1^ [32]. It should be noted that this flux indirectly depends on the buffering capacity of the cytoplasm, because *D* decreases together with *f*.

Assuming that the volume of the shell is 1/10 of the volume of the cytosol, the corresponding change of Ca^2+^ concentration in the cytosol (*Ca_cyt_*) is given by $-J_{diff}/9$.

The cytosolic compartment also exchanges Ca^2+^ with the ER, in which the Ca^2+^ concentration is much larger, around 500 μM. Ca^2+^ exchanges between these two compartments have been much studied, both experimentally and by modelling, because they can produce cytosolic Ca^2+^ oscillations [19,33]. It is known that relative changes in Ca^2+^ concentration are much smaller in the ER than in the cytoplasm. In the present study where only basal, non-stimulated Ca^2+^ release from the ER is simulated, we consider that the ER Ca^2+^ concentration remains constant [34,35] and fixed Ca_ER_ at 500 μM [36]. Basal Ca^2+^ efflux from the ER is given by

(6) $$J_{ER}=k_{in}\left(Ca_{ER}-Ca_{cyt}\right)$$

in which *k_in_* gathers basal ER Ca^2+^ release through the IP_3_R and the RyR, as well as unspecific leaks. The opposite flux, i.e., Ca^2+^ pumping from the cytosol to the ER, is mediated by the sarco- or endoplasmic reticulum Ca^2+^ ATPase (SERCA) and is modelled as

(7) $$J_{SERCA}=V_{SERCA}\frac{Ca_{cyt}^{2}}{K_{SERCA}^{2}+Ca_{cyt}^{2}}$$

in which *V_SERCA_* and *K_SERCA_* stand for the maximal velocity and the half saturation constant of SERCA, respectively.

When considering these additional processes, the core model originally described by Equations (1) and (2) takes the form:

(8) $$C_{m}\frac{dV}{dt}=-I_{Na\left(V\right)}-I_{K\left(V\right)}-I_{Ca\left(V\right)}-I_{K\left(Ca\right)}-I_{NCX}+I_{Inj}$$

(9) $$\frac{dCa}{dt}=f\left[\frac{-I_{Ca\left(V\right)}+2I_{NCX}}{2FV_{shell}}-J_{PMCA}\right]-J_{diff}$$

(10) $$\frac{dCa_{cyt}}{dt}=\frac{J_{diff}}{9}+k_{in}\left(Ca_{ER}-Ca_{cyt}\right)-J_{SERCA}$$

Together with the equations for the currents (Equations (S1)–(S4)) and for the fluxes (Equations (3)–(7)), the evolution Equations (8)–(10) define the model used throughout the whole study. The values of parameters are given in Table 1, unless indicated in the figure legends. Numerical integration and bifurcation diagrams have been obtained using XPPAUT and its AUTO package [37]. The code is available on github (https://github.com/genedupont/amyloids, accessed on 26 November 2021).

## 3. Results

### 3.1. Model Behavior in the Absence of Aβ Peptides

**Figure 2:**
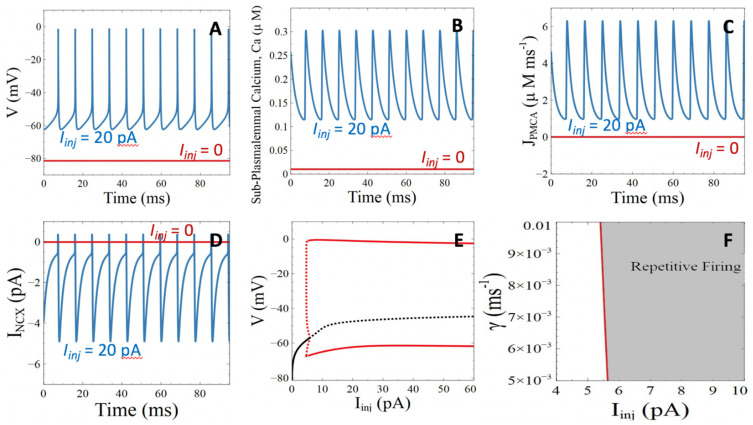
Analysis of the behavior of the model in the absence of perturbation by Aβ peptides. (**A**) Evolution of voltage in the absence of the injected current (red line) or when *I_Inj_* = 20 pA (blue line). (**B**) Evolution of the Ca^2+^ concentration in the subplasmalemmal compartment in the absence of the injected current (red line) or when *I_Inj_* = 20 pA (blue line). (**C**) Ca^2+^ fluxes through the PMCA in the absence of injected current (red line) or when *I_Inj_* = 20 pA (blue line). (**D**) Ca^2+^ fluxes through the NCX in the absence of injected current (red line) or when *I_Inj_* = 20 pA (blue line). (**E**) Bifurcation diagram showing the steady state solutions of the model for increasing values of *I_inj_.* Plain and dashed curves correspond to stable and unstable situations, respectively. Black curves indicate steady states and red ones, oscillatory solutions, with the high and low curves showing the maximal and minimal values of membrane voltage reached during repetitive action potentials. (**F**) Two-parameter bifurcation diagram showing the zone of stability of the steady state (in white) and of the oscillatory solutions (in grey) in the (*I_inj_*, *γ*) plane. Thus, a faster diffusion of Ca^2+^ in the cytosol decreases the threshold value of current that must be injected into the cell to induce electrical activity.

We first checked if the extended model described in Section 2 provides an adequate description of the neuronal activity in unperturbed conditions [2,29]. For a sufficiently large value of the injected current, *I_inj_*, the model displays repetitive action potentials (Figure 2A), which are accompanied by periodic variations of the Ca^2+^ concentration in the sub-plasmalemmal compartment (*Ca*, Figure 2B). These changes in *Ca* are due to the Ca^2+^ entry through the voltage sensitive Ca^2+^ channels ($I_{Ca\left(V\right)})\;$ and extrusion from this compartment via three mechanisms. The most significant is the PMCA (Figure 2C), with a flux that reaches ~6 μMms^−1^. The current mediated by NCX reaches, at most, −5 pA (Figure 2D), which corresponds to a Ca^2+^ flux of about 0.1 μMms^−1^. These values are in the order of experimental observations [38]. The third mechanism by which Ca^2+^ leaves the sub-plasmalemmal Ca^2+^ compartment is diffusion into the cytoplasm. This term oscillates between 5 × 10^−7^ and 2.6 × 10^−6^ μMs^−1^. Such modest fluxes are due to the small value of γ (Equation (5)), which reflect the slow diffusion of Ca^2+^ in the cytoplasm because of buffering. Because sub-plasmalemmal Ca^2+^ is mainly removed by the PMCA, when the cell is electrically active the Ca^2+^ concentration in the cytoplasm remains practically constant at 40 nM. This value of *Ca_cyt_* is determined by the exchanges with the ER.

The bifurcation diagram (Figure 2E) of the extended model resembles that of the original model. After a threshold value of injected current, the stable steady state (plain black line) loses its stability and action potentials (red lines) develop. The Hopf bifurcation is sub-critical, but the zone of coexistence between the stable steady state and the stable limit cycle is very limited [22]. Ca^2+^ diffusion potentially affects the firing threshold of the injected current (Figure 2F). When diffusion is fast, *I_inj_* at the bifurcation point decreases. Indeed, Ca^2+^ entering the cell is removed more efficiently, reducing the activation of the K(Ca) current. Membrane polarization is therefore attenuated, which decreases the value of the depolarizing current that must be injected to initiate an action potential.

The model described in Section 2 provides a simple and realistic description of neuronal and Ca^2+^ dynamics. In the following, we use this model to investigate how changes in Ca^2+^ dynamics influence neuronal excitability. In particular, we focus on how known targets of Aβ peptides modify (1) the threshold value of injected current triggering repetitive action potentials, i.e., the value of *I_inj_* at the bifurcation point, and (2) the frequency of repetitive APs. A decrease of the threshold value of *I_inj_* or an increase in frequency from the control situation (Figure 2E) will be interpreted as neuronal hyperexcitability.

### 3.2. Aβ Peptides Induced Changes in the Rates of Ca^2+^ Pumps and Pores

#### 3.2.1. Ca^2+^ ATPases

**Figure 3:**
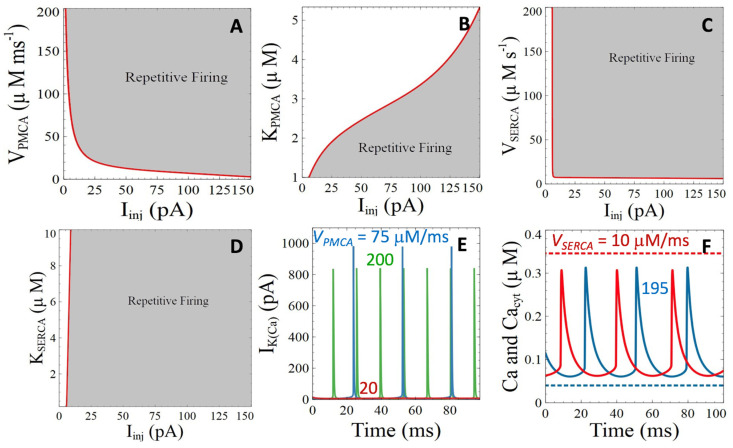
Simulations of the impacts of Aβ peptides induced perturbations of PMCA’s and SERCA’s on neuronal activity and Ca^2+^ dynamics. (**A**,**B**) Two parameter bifurcation diagrams showing the zone of existence of neuronal activity when changing the maximal rate (**A**) or the half-saturation constant (**B**) of the PMCA. (**C**,**D**) Two parameter bifurcation diagrams showing the zone of existence of neuronal activity when changing the maximal rate (**C**) or the half-saturation constant (**D**) of the SERCA. (**E**) Temporal evolution of *I_K*(*Ca*)*_* for *I_Inj_* = 5.83 pA and *V_PMCA_* = 20 μMms^−1^ (red curve), 75 μMms^−1^ (blue curve) and 200 μMms^−1^ (green curve). (**F**) Temporal evolution of the Ca^2+^ concentrations for *I_Inj_* = 5.83 pA when changing the maximal rate of the SERCA. Plain lines correspond to Ca^2+^ concentrations in the subplasmalemmal space for *V_SERCA_* = 10 μMms^−1^ (blue curve) and 195 μMms^−1^ (purple curve), showing the lack of influence of the SERCA pump activity on Ca^2+^ concentration just below the plasma membrane during electrical activity. Corresponding values of the Ca^2+^ concentration in the bulk of the cytoplasm are indicated by dashed lines (red curve, *V_SERCA_* = 10 μMms^−1^; blue curve, *V_SERCA_* = 195 μMms^−1^).

PMCA are dysregulated in AD in an Aβ-dependent manner [39]. The potential influence of this dysregulation deserves investigation, as we have seen in the previous section that active pumping of Ca^2+^ out of the cell by the PMCA plays the most important role in the extrusion of Ca^2+^ from the sub-plasmalemmal space (Figure 2). Thus, changes in the kinetic parameters of the PMCA are expected to have a strong influence on the sub-membrane Ca^2+^ dynamics and hence on neuronal activity. A decrease in the maximal rate of the PMCA (*V_PMCA_*) indeed provokes an increase in the amplitude of the current that must be injected to initiate neuronal firing (Figure 3A). Simulations indicate that the increase of *I_K_*_(*Ca*)_ with decreasing PMCA activity is responsible for the observed change in *I_inj_* at the bifurcation point. Figure 3E shows that the *I_K_*_(*Ca*)_ current gets larger when *V_PMCA_* decreases. Below a threshold value (red line in Figure 3A), action potentials disappear. Changing the half saturation constant of the PMCA from its default value (*K_PMCA_* = 1 μM) also affects the domain of repetitive firing (Figure 3B), although in a less abrupt manner.

The strong influence of PMCA on electrical activity contrasts with the lack of effect of changing the kinetic parameters of the SERCA visible in Figure 3C,D. It can be seen that the value of the injected current leading to repetitive action potentials is not influenced by the values of *V_SERCA_* and *K_SERCA_*. Although changing these parameters modifies the level of Ca^2+^ in the cytoplasm, the influence of this change on sub-plasmalemmal Ca^2+^, and hence on electrical activity, is nearly nil, as shown in Figure 3F. Thus, changes in the activity of PMCA and SERCA pumps have a strong influence on the concentrations of Ca^2+^ in the sub-plasmalemmal compartment and in the bulk of the cytosol, respectively. These changes are however not transmitted to the other compartment because the transport of Ca^2+^ by active pumping by far exceeds diffusion. It should be kept in mind that the average cytosolic Ca^2+^ concentration, which corresponds to (*Ca*+9*Cac*)/10, is influenced in both cases. As far as neuronal activity is concerned, excitability decreases together with the activity of the PMCA’s, while it is not influenced by the SERCAs.

#### 3.2.2. Ryanodine Receptor- and IP_3_ Receptor-Mediated Basal Release of ER Ca^2+^

**Figure 4:**
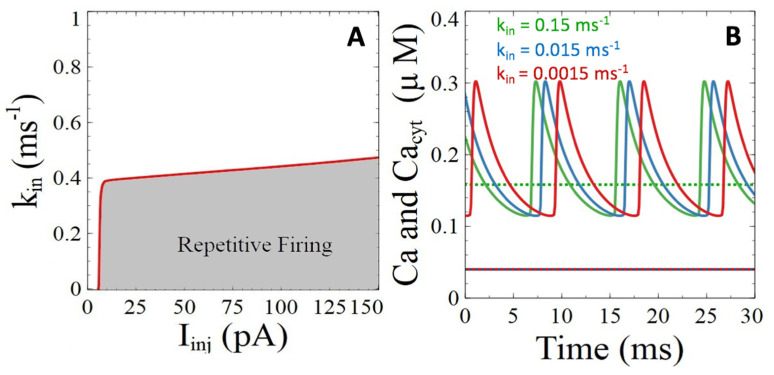
Simulations of the impacts of Aβ peptides induced perturbations of the basal rate of Ca^2+^ release from the ER on neuronal activity and Ca^2+^ dynamics. (**A**) Two parameter bifurcation diagram showing the zone of existence of neuronal activity when changing the rate constant of Ca^2+^ release from the ER. (**B**) Temporal evolution of the Ca^2+^ concentrations for *I_Inj_* = 20 pA when changing the rate constant *k_in_*. Plain lines correspond to Ca^2+^ concentrations in the subplasmalemmal space for *k_in_* = 0.15 ms^−1^ (green curve), 0.015 ms^−1^ (blue curve), and 0.0015 ms^−1^ (red curve). Corresponding values of the Ca^2+^ concentration in the bulk of the cytoplasm are indicated by dashed lines.

In neurons, Ca^2+^ is released from the ER via ryanodine and IP_3_ receptors and there is compelling evidence that Aβ peptides mobilize internal Ca^2+^ via these two receptors [40]. Moreover, it has been shown that the injection of Aβ42 oligomers stimulates the production of IP_3_ [41]. Thereby, Aβ peptides not only exacerbate stimulus-induced cytosolic Ca^2+^ responses, but also provoke a sustained increase in the basal rate of ER Ca^2+^ release that can be modeled by an increase in the value of parameter *k_in_* that appears in Equation (6). Bifurcation analysis of the influence of this parameter (Figure 4A) indicates that it does not affect the PM membrane electrical activity up to a point where cytosolic Ca^2+^ concentration becomes so high that the cell can only be electrically silent. However, this situation is not appropriately represented by the present model, which does not take the Ca^2+^-induced Ca^2+^ release regulation of these two receptors into account. For more modest changes in *k_in_*, the conclusions are qualitatively the same as for the SERCA pumps, pointing to an independence between cytoplasmic Ca^2+^ changes on one hand and sub-plasmalemmal Ca^2+^ and electrical activity on the other hand. That the increase in cytosolic Ca^2+^ does barely not affect sub-membrane Ca^2+^ is visible in Figure 4A,B.

Simulations thus predict that an increase in the rate of basal Ca^2+^ release from the ER does not change the levels of Ca^2+^ in the subplasmalemmal space, and thus also not neuronal excitability.

#### 3.2.3. Pores of β-Amyloids in the Plasma Membrane

Aβ peptides can form Ca^2+^ permeable pores in the PM [28,41]. We investigated the effect of such pores in the model defined in Section 2 by considering the following rate of Ca^2+^ entry in the sub-plasmalemmal compartment:

(11) $$J_{Abeta}=V_{Abeta}\frac{1}{1+e^{\left(V-q_{1}\right)/q_{2}}}$$

which reflects a passive, electrogenic flux [42]. *V_Abeta_*, the maximal rate of Ca^2+^ entry through the pores, reflects both the single pore permeability and the number of pores in the PM. *q*_1_ and *q*_2_ characterize the voltage-dependence of Ca^2+^ entry through the pores. It reproduces a steep decrease of the flux of positively charged Ca^2+^ ions through the pores when the membrane voltage increases.

The evolution equations for sub-plasmalemmal Ca^2+^ and membrane voltage are modified to consider this flux, i.e.,

(12) $$\frac{dCa}{dt}=f\left[\frac{-I_{Ca\left(V\right)}+2I_{NCX}}{2FV_{shell}}-J_{PMCA}+J_{Abeta}\right]-J_{diff}$$

(13) $$C_{m}\frac{dV}{dt}=-I_{Na\left(V\right)}-I_{K\left(V\right)}-I_{Ca\left(V\right)}-I_{K\left(Ca\right)}-I_{NCX}+I_{Inj}+2FV_{Shell}\;J_{Abeta}$$

where *F* stands for the Faraday constant and *V_shell_* for the volume of the sub-plasmalemmal compartment, as defined above.

**Figure 5:**
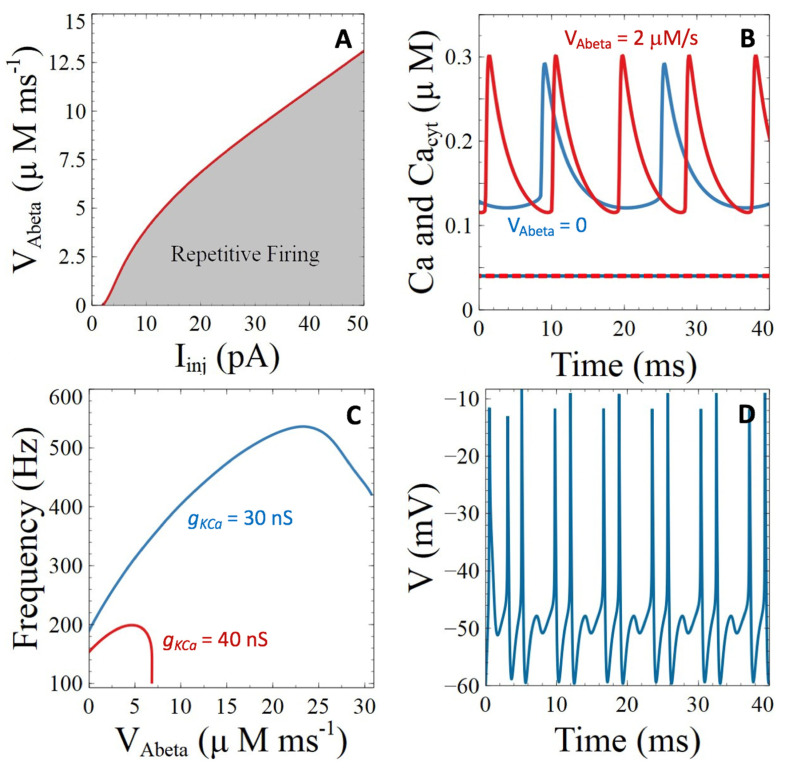
Simulations of the impacts of the presence of pores of Aβ peptides in the plasma membrane on neuronal activity and Ca^2+^ dynamics. (**A**) Two parameter bifurcation diagram showing the zone of existence of neuronal activity when changing the rate constant of Ca^2+^ entry through the pores. (**B**) Temporal evolution of the Ca^2+^ concentrations for *I_Inj_* = 20 pA and *g_KCa_* = 30 nS in the absence (blue curve) or presence (red curve, *V_Abeta_* = 2 μMms^−1^) of pores of Aβ peptides. Plain lines indicate Ca^2+^ concentration in the subplasmalemmal compartment and dashed ones in the bulk of the cytosol. (**C**) Relation between the frequency of action potentials and the value of *V_Abeta_* for *I_Inj_* = 20 pA and *g_KCa_* = 40 (red curve) or 30 nS (blue curve). (**D**) Irregular electrical activity occurring for large values of *V_Abeta_*. *I_Inj_* = 20 pA, *V_Abeta_* = 20, and *g_KCa_* = 30 nS.

Simulations of the model predict that for increasing values of *V_Abeta_*, larger currents must be injected to induce a repetitive action potential (Figure 5A). Again, this is due to the increase in subplasmalemmal Ca^2+^ concentration (Figure 5B) that stimulates the Ca^2+^-dependent K^+^ current, which hyperpolarizes the cell. Ca^2+^ concentration in the cytosol is not affected (dashed lines in Figure 5B). These consequences of the presence of pores of amyloids in the plasma membrane are qualitatively similar to the Aβ peptides induced reduction of PMCA activity. However, these two targets differ by the fact that a flux of Ca^2+^ through the pores induces a change in membrane voltage (Equation (13)), while PMCAs are electroneutral [31]. This difference is responsible for the possible increase in the frequency of repetitive AP’s with *V_Abeta_* (Figure 5C) that is not observed upon PMCA inhibition. Thus, although the presence of pores increases the threshold value of current that must be injected to induce neuronal repetitive firing, once active, the frequency is higher in the presence of Aβ peptide pores. As expected intuitively, the spiking frequency increases faster with *V_Abeta_* if the conductance of the Ca^2+^-activated K^+^ channels decreases (compare blue and red curve in Figure 5C). When *V_Abeta_* is very large, the shape of the action potential is modified drastically and irregular spiking occurs (Figure 5D).

Thus, ions permeable pore of Aβ peptides provoke an increase in sub-plasmalemmal Ca^2+^. Because this influx is associated with PM depolarization, it can lead to an increase in spiking frequency for moderate values of the conductance of the K(Ca) channels. However, the threshold value of injected current is not affected by the pores.

### 3.3. Aβ Peptides Induced Changes in Voltage-Sensitive Ca^2+^ Currents

#### 3.3.1. Voltage-Gated Ca^2+^ Channels

**Figure 6:**
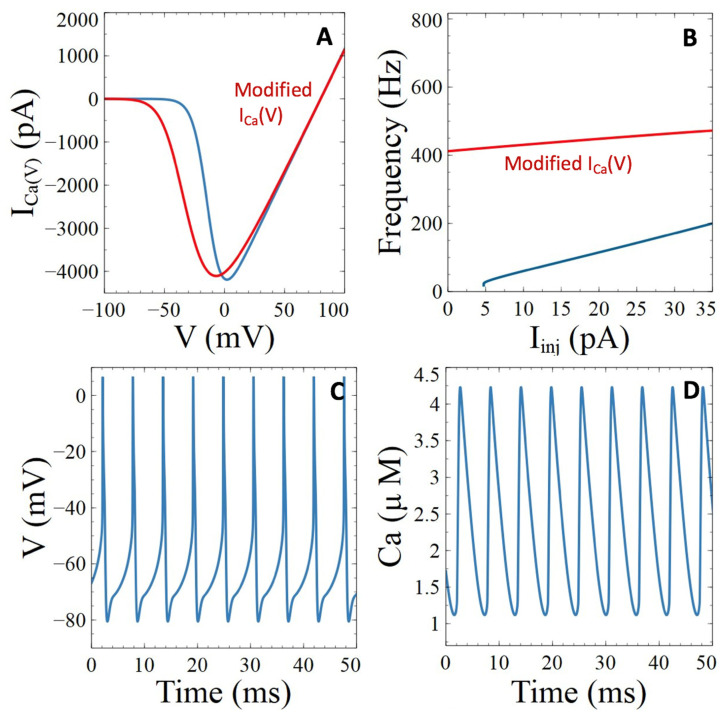
Simulations of the impacts of Aβ peptide induced perturbations of the voltage-gated Ca^2+^ channels on neuronal activity and Ca^2+^ dynamics. (**A**) Changes in the values of parameters involved in the activation and deactivation of the voltage gated Ca^2+^ channel, as described in Equations (14) and (15), allows for reproducing the Aβ peptide induced shift in the voltage dependency described by Ishii et al. (2019) [45]. The blue and red curves correspond to the default and modified values of parameters, respectively. Curves have been obtained by solving Equations (14), (15), (S7), (S18) and (S19) at steady state. (**B**) Comparison of the frequency vs. injected current relations for the default (blue) and modified values of parameters of the voltage-gated Ca^2+^ channel. (**C**,**D**) Temporal evolution of membrane voltage and subplasmalemmal Ca^2+^ concentrations for *I_Inj_* = 20 pA with the values of parameters corresponding to the voltage gated Ca^2+^ channel modified by the presence of Aβ peptides.

The L-type Ca^2+^ channel has attracted much attention in the field of Alzheimer’s disease. Over-activation of these channels by Aβ peptides has been observed in rat cultured cortical and hippocampal neurons [43]. Injection of Aβ peptides upregulates L-type Ca^2+^ channels in rat hippocampal neurons [25], a phenomenon that is also observed in AD transgenic mice [44]. In particular, the maximal activation of these channels is changed from 0 to about −15 mV in the presence of Aβ [45]. Such a change can be modelled by modifying the values of parameters in the activation and inactivation functions of the L-type Ca^2+^ current, noted α_s_ and β_s_ in Equations (S18) and (S19). When considering

(14) $$\alpha_{S}=\frac{150}{1+exp^{-0.072\left(V-5\right)}}$$

and

(15) $$\beta_{S}=\frac{0.1\left(V+8.9V\right)}{exp^{0.02\left(V+8.9\right)}-1}$$

instead of the default function defined by Equations (S18) and (S19), the maximal current indeed corresponds to a ~−15 mV voltage, as shown in Figure 6A.

Simulations indicate that repetitive APs occur in the absence of injected current with these modified values of parameters for the L-type channel (Figure 6B). Moreover, for a given value of *I_inj_*, the frequency of action potentials is larger than in the control situation. As can be seen in Figure 6C, APs become significantly broader, which results in high levels of Ca^2+^ in the sub-plasmalemmal compartment (Figure 6D). As for the preceding Aβ peptides targets, these changes do not significantly affect the Ca^2+^ level in the bulk of the cytoplasm, because most of this Ca^2+^ is extruded to the extracellular medium by the PMCA and the NCX (not shown).

Thus, Aβ peptides induced up-regulation of the L-type Ca^2+^ channel results into marked neuronal hyperexcitability. This is reflected by an increase in both the firing frequency and the propensity of the cell to become electrically active. It also significantly raises the concentration of the sub-plasmalemmal Ca^2+^ during activity.

#### 3.3.2. Ca^2+^-Sensitive Potassium Channels

The activity of the Ca^2+^-sensitive potassium channels has also been shown to be modified by the presence of Aβ peptides [26]. In mouse neurons, intracellular injection of these amyloids suppresses BK channels [46]. The same effect can be observed in cortical pyramidal cells from 3xTg mice [47]. More indirectly, Sosulina et al. (2020) [11] observed an increased intrinsic excitability of CA1 neurons in the absence of synaptic changes in hippocampal slices from a rat model of AD at the early stages following Aβ deposition. Because of the simultaneous decrease in the resting membrane potential and increase in AP half-width, this increase in intrinsic excitability was hypothesized to originate from a decrease in the activity of the K(Ca) channels.

**Figure 7:**
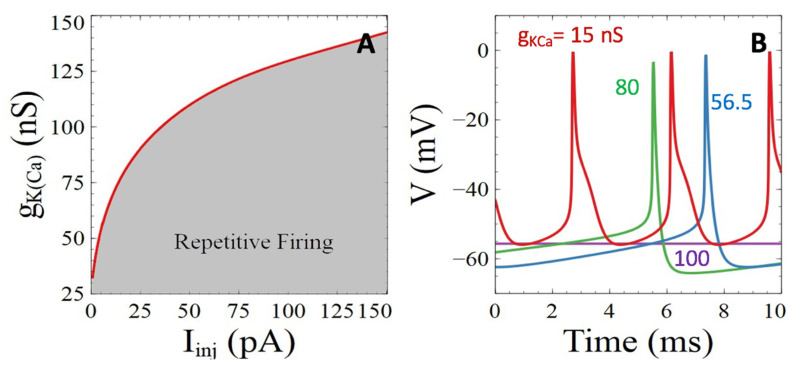
Simulations of the impacts of Aβ peptide induced perturbations of the Ca^2+^ -activated K^+^ channels on neuronal activity and Ca^2+^ dynamics. (**A**) Two parameter bifurcation diagram showing the zone of existence of neuronal activity when changing the conductance of the the Ca^2+^ activated K^+^ channels. (**B**) Simulated evolution of membrane voltage for *I_inj_* = 20 pA and *g_KCa_* = 15 (red), 56.5 (blue), 80 (green), and 100 nS (purple).

In agreement with these observations, a decrease in the conductance of the K(Ca) channels provokes a marked decrease in the threshold value of current that must be injected to induce repetitive firing (Figure 7A). As visible in Figure 5C, the frequency of repetitive APs decreases if K(Ca) channels are less active, because they have a marked hyperpolarizing effect that delays the onset of the next spike. The minimal value of voltage between two spikes indeed decreases with the value of *g_K(Ca)_* (Figure 7B). Simulations also show that the half-width of APs increases with decreasing values of *g_K(Ca)_* (Figure 7B), as observed in the neurons where these channels are down-regulated by Aβ peptides.

Thus, a reduction in the conductance of K(Ca) points to electrical hyperexcitability at all levels: reduction of the threshold current intensity required to induce repetitive AP’s, and an increase in the frequency and broadening of the AP’s.

## 4. Discussion

Mathematical modeling provides a unique tool to derive insight into the possible molecular mechanisms that may be relevant in AD. We therefore used a modeling approach to systematically investigate the various effects of Aβ peptides on calcium dynamics and neuronal excitability. We extended a previously proposed mathematical description of the electrical activity of cerebellar granule cells [2] to propose a generic minimal model of neuronal and calcium dynamics. To take spatial heterogeneity into account, we considered a compartmental model, which is a standard approach that is largely used to model Ca^2+^ microdomains, for example [19].

Our simulations point to a clear decoupling between the evolution of Ca^2+^ concentration just below the PM and in the bulk of the cytosol. Indeed, Ca^2+^ changes in the sub-plasmalemmal compartment are governed by Ca^2+^ exchanges with the extracellular medium that occur with a much larger rate than passive diffusion from and to the bulk of the cytosol. On the other hand, these changes have a moderate influence on the Ca^2+^ concentration in the much larger cytosolic space. In indirect agreement with this independence between sub-plasmalemmal and bulk cytosolic Ca^2+^, Yao et al. (2020) [48] reported that limiting RYR open time does not have a major effect on the afterhyperpolarization current in hippocampal CA1 neurons, which agrees with the lack of effect of raising the bulk cytoplasmic Ca^2+^ on the activity of a Ca^2+^-dependent voltage gated channel located in the PM. In the context of AD, the decoupling between membrane and bulk cytosolic Ca^2+^ observed in the simulations suggests that disruptions of Ca^2+^ homeostasis that interfere with signaling and promote the progression of the disease are unrelated from changes in the electrical properties of the neurons. It should be kept in mind, however, that this conclusion only holds at the single cell level. At the network level, Ca^2+^ could affect electrical activity because AD-related synaptic loss may be partly due to Ca^2+^ dysregulations [49].

As a direct consequence of the decoupling between sub-PM and bulk cytosolic Ca^2+^, our results predict that the reported modifications induced by Aβ peptides on the activities of transporters affecting deep cytosolic Ca^2+^ have no impact on neuronal excitability in the absence of the ER-Ca^2+^ mobilizing agonist. The fact that we considered conditions corresponding to the absence of stimulation of IP_3_ or RyR receptors is in contrast with the approaches followed by Latulippe et al. [13] and Liu et al. [14]. In the two studies, devoted to electrically non-excitable cells, a homogeneous model was considered to analyze the impact of Aβ peptides on Ca^2+^ dynamics. They concluded that amyloids affect the existence, frequency, and shape of agonist induced Ca^2+^ oscillations. The latter conclusion is significant in view of the observation that Aβ oligomers can stimulate IP_3_ production [41], a point that is not investigated in our present study. The impact of this effect of amyloids on the changes in cytosolic Ca^2+^ and consequently on mitochondrial metabolism has also been investigated by modelling [35].

In contrast, when Aβ peptides affect Ca^2+^ fluxes across the PM, they have a significant influence on the membrane neuronal activity in the model. It was shown that Aβ peptides bind to the catalytic site of the PMCA, which corresponds to an increase in the value of *K_PMCA_* appearing in Equation (3). As shown in Figure 3B, this induces a reduction of neuronal excitability in the model. Aβ peptides also tend to increase sub-membrane Ca^2+^ by forming pores in the PM. In the model, they have rather mixed consequences on neuronal activity (Figure 4). On the one hand, they increase the threshold value of current that must be injected in the neuron to induce repetitive action potential. On the other hand, they can provoke an increase in the spiking frequency. While the latter observation is associated with hyperexcitability, the former is associated with hypo-excitability. These apparently opposite consequences can be ascribed to the fact that the entry of Ca^2+^ through the pores stimulates the hyperpolarizing K(Ca) current, but also depolarizes the cell membrane. Indeed, hyperexcitability is more pronounced when decreasing the conductance of the K(Ca) channel. In addition, if Ca^2+^ influx through the pores is very large, aberrant spiking in the form of complex neuronal activity can also be observed in the model. As a perspective, it would be interesting to test if the same simulated behaviors can be recovered when considering a stochastic Markov model validated against experimental data [28] to describe the dynamics of pore opening and closing. Finally, the reported changes in the activities of L-type Ca^2+^ channels and Ca^2+^-sensitive K^+^ channels in the presence of Aβ peptides clearly point to hyperexcitability when considered in the model. These results are in agreement with the numerous studies reporting neuronal hyperexcitability in the presence of Aβ peptides. In particular, limiting the open time duration of RYR2 in the hippocampal CA1 neurons of an AD mouse model upregulates the surface expression of the K(Ca) channel and prevents electrical hyperactivity, but does not directly modulate the activity of the K(Ca) channels [48]. Altogether, our simulation results indicate that the reported consequences of Aβ peptides induced modifications of Ca^2+^ fluxes or Ca^2+^-regulated currents have qualitatively different outcomes. However, it should be noted that although Aβ peptides are most of the time associated with neuronal hyperexcitability, some studies however report opposite consequences [50,51].

The seemingly contradictory effects of Aβ peptides reported in this study may also be due to the limitations of the proposed model. For example, it does not consider the specificities of the various neuronal cell types. Additionally, we considered one target of Aβ peptides at a time, which is important to dissect the pathological consequences of the oligomers of amyloids, but does not correspond to a realistic situation. Because they are limited to a single neuron, simulations do not include network effects, which play a crucial role in the cognitive defects related to the disease [52]. It is clear that simplified modelling, such as the one performed here, does not provide a faithful view of the complexity of calcium signaling and neuronal activity corresponding to an in vivo situation. However, it allows for drawing some simple and unambiguous conclusions, which can help analyzing experimental observations when considered from a larger perspective.

## Figures and Tables

**Table 1:** List of the parameters of the model. Values indicated have been used for all figures, except if mentioned in the legend. Parameters associated with the dynamics of the currents are given directly in the equations listed in the Supplementary Material.

Parameter Name	Parameter Meaning	Value
$C_{m}$	Membrane capacity	3.14 pF
$V_{\mathrm{shell}}$	Volume of the shell	$26.378\;{\mu m}^{3}$
F	Faraday constant	$96.485\;{C\;\mathrm{mol}}^{-1}$
f	Calcium buffering capacity of the cytoplasm	0.01
$I_{\mathrm{inj}}$	Injected Current	[0.150] pA
$k_{\mathrm{in}}$	$\mathrm{Rate}\;\mathrm{constant}\;\mathrm{of}\;\mathrm{unstimulated}\;Ca^{2+}$ release from the ER	$0.015\;{\mathrm{ms}}^{-1}$
${\mathrm{Ca}}_{\mathrm{ER}}$	$\mathrm{ER}\;{\mathrm{Ca}}^{2+}$ concentration	500 μM
γ	Shell-Cytoplasm Diffusion Coefficient	$0.001{\;\mathrm{ms}}^{-1}$
$V_{{\mathrm{Na}}^{+}}$	$\mathrm{Equilibrium}\;\mathrm{Potential}\;\mathrm{for}\;{\mathrm{Na}}^{+}$ ions	55 mV
$V_{K^{+}}$	$\mathrm{Equilibrium}\;\mathrm{Potential}\;\mathrm{for}\;K^{+}\;$ ions	−90 mV
$V_{{\mathrm{Ca}}^{2+}}$	$\mathrm{Equilibrium}\;\mathrm{Potential}\;\mathrm{for}\;Ca^{2+}$ ions	80 mV
$V_{\mathrm{NCX}}$	Equilibrium Potential for the NCX channel	−20 mV
$g_{{\mathrm{Na}}^{+}}$	$\mathrm{Conductance}\;\mathrm{of}\;\mathrm{the}\;\mathrm{voltage-gated}\;{\mathrm{Na}}^{+}$ channel	172 nS
$g_{K^{+}}$	$\mathrm{Conductance}\;\mathrm{of}\;\mathrm{the}\;\mathrm{voltage-gated}\;K^{+}$ channel	28 nS
$g_{{\mathrm{Ca}}^{2+}}$	$\mathrm{Conductance}\;\mathrm{of}\;\mathrm{the}\;\mathrm{voltage-gated}\;{\mathrm{Ca}}^{2+}$ channel	58 nS
$g_{K-{\mathrm{Ca}}^{2+}}$	$\mathrm{Conductance}\;\mathrm{of}\;\mathrm{the}\;K^{+}$$\mathrm{activated}\;{\mathrm{Ca}}^{2+}$ channel	56.5 nS
$K_{\mathrm{NCX}}$	Half saturation constant of the NCX channel	1 μM
$K_{\mathrm{PMCA}}$	Half saturation constant of the PMCA	1 μM
$K_{\mathrm{SERCA}}$	Half saturation constant of the SERCA	0.2 μM
$V_{\mathrm{PMCA}}$	Maximal velocity of the PMCA	$75\;{\mu M\;\mathrm{ms}}^{-1}$
$V_{\mathrm{SERCA}}$	Maximal velocity of the SERCA	$195\;{\mu M\;\mathrm{ms}}^{-1}$
$V_{\mathrm{Abeta}}$	$\mathrm{Maximal}\;\mathrm{rate}\;\mathrm{of}\;{\mathrm{Ca}}^{2+}$ release through pores of Aβ peptides	$0\;{\mu M\;\mathrm{ms}}^{-1}$
$q_{1}$	$\mathrm{Voltage}\;\mathrm{dependence}\;\mathrm{of}\;{\mathrm{Ca}}^{2+}$ entry through the pores of Aβ peptides	−30 mV
$q_{2}$	$\mathrm{Voltage}\;\mathrm{dependence}\;\mathrm{of}\;{\mathrm{Ca}}^{2+}$ entry through the pores of Aβ peptides	23 mV

## Data Availability

The code of the model is available at https://github.com/genedupont/amyloids (accessed on 26 November 2021).

## Supplementary Materials

The following are available online at https://www.mdpi.com/article/10.3390/cells11040615/s1, Additional equations of the model.
