# Supplementary material for: Impact of β-Amyloids Induced Disruption of Ca2+ Homeostasis in a Simple Model of Neuronal Activity
Source: Cells. 2022 Feb 10;11(4):615. doi: 10.3390/cells11040615 (PMC8869902; doi:10.3390/cells11040615)
Supplement: Supplementary file 1 [file cells-11-00615-s001.zip › cells-1504952-supplementary.pdf]

Supplementary Material: Impact of β-amyloids induced disruption of Ca2+ homeostasis in a simple model of neuronal activity

Francisco Prista Santos von Bonhorst Silva, David Gall and Geneviève Dupont

All the currents are modelled using the model and gating variables. The values of these currents are given by the following equations:

$$I_{Na^+(V)} = \overline{g}_{Na^+} m_\infty^3 h \left(V - V_{Na^+}\right) \tag{S.1}$$

$$I_{K^+(V)} = \overline{g}_{K^+} n_\infty^4 \left(V - V_{K^+}\right) \tag{S.2}$$

$$I_{Ca^{2+}(V)} = \overline{g}_{Ca^{2+}} s^2 \left(V - V_{Ca^{2+}}\right) \tag{S.3}$$

$$I_{K^+(Ca)} = \overline{g}_{Ca^{2+}-K^+} a \left(V - V_{K^+}\right) \tag{S.4}$$

where m, h, n, s, a are gating variables for their respective currents as discussed before, V_{ion} is the equilibrium potential as determined by Nernst's equation for a certain ion species, the $\overline{g}$'s are the constants and the indices "∞" indicate that we take the stationary values for the corresponding gating variables. Using the simplification we talked about in Chapter(**??**) Eq(**??**), we obtained the following equations:

$$\frac{dh}{dt} = \frac{h_\infty(V) - h(V)}{\tau_h(V)} \tag{S.5}$$

$$\frac{ds}{dt} = \frac{s_\infty(V) - s(V)}{\tau_s(V)} \tag{S.6}$$

$$\frac{da}{dt} = \frac{a_\infty(V, Ca) - a(V, Ca)}{\tau_a(Ca, V)} \tag{S.7}$$

Notice, that both the gating variables, the stationary states of these and the time-scales depends on V, with the notable exception of the gating variable "a" associated to the activated channels, which also depends on the concentration as expected.

The remaining equations for gating variables and timescales are given by:

$$m_\infty = [1 + \exp(-0.147(V + 39))]^{-1} \tag{S.8}$$
$$h_\infty = [1 + \exp(-0.178(V + 50))]^{-1} \tag{S.9}$$
$$n_\infty = [1 + \exp(-0.091(V + 38))]^{-1} \tag{S.10}$$
$$s_\infty = \frac{\alpha_s}{\alpha_s + \beta_s} \tag{S.11}$$
$$a_\infty = \frac{\alpha_a}{\alpha_a + \beta_a} \tag{S.12}$$
$$\tau_h = max(0.045, 0.6[\exp(-0.089(V + 50)) + \exp(0.089(V + 50))]^{-1}) \tag{S.13}$$
$$\tau_s = \frac{1}{\alpha_s + \beta_s} \tag{S.14}$$
$$\tau_a = \frac{1}{\alpha_a + \beta_a} \tag{S.15}$$
$$\alpha_s = \frac{8}{1 + \exp(-0.072(V - 5))} \tag{S.16}$$
$$\beta_s = \frac{0.1(V + 8.9)}{\exp(0.2(V + 8.9)) - 1} \tag{S.17}$$
$$\alpha_a = \frac{12.5}{1 + \frac{0.15\exp(-0.085V)}{[Ca]}} \tag{S.18}$$
$$\beta_a = \frac{7.5}{1 + \frac{[Ca]}{0.015\exp(-0.077V)}} \tag{S.19}$$

Where the concentrations are in μM, the potential in mV and the timescales in ns.
